# Generative AI as a cognitive co-learner: a developmental framework for AI literacy in health sciences education

**DOI:** 10.3389/frai.2026.1871363

**Published:** 2026-08-10

**Authors:** Myo Zin Oo, Soe Sandi Tint, Kyi Phyu Myo Khaing, Thanakit Noithanom, Ammar Almurisi Bin Abdul Hamid, Myo Khaing Oo, Hlaing Thaw Dar, Rattanathorn Intarak

**Affiliations:** 1 Research Institute for Health Sciences, Chiang Mai University, Chiang Mai, Thailand; 2 Global Health and Chronic Conditions Research Center, Chiang Mai University, Chiang Mai, Thailand; 3 Department of Family Medicine, Faculty of Medicine, Chiang Mai University, Chiang Mai, Thailand; 4 Unity Concord International School, Chiang Mai, Thailand; 5 Myanmar Development Prosperity Initiative, Taunggyi, Myanmar; 6 Faculty of Public Health, St Teresa International University, Nakhon Nayok, Thailand; 7 School of Medicine, IMU, Kuala Lumpur, Malaysia; 8 Independent Researcher, Yangon, Myanmar; 9 Public Health and Community Medicine Department, School of Medicine, IMU, Kuala Lumpur, Malaysia

**Keywords:** AI literacy, calibrated trust, cognitive co-learner, generative artificial intelligence, health sciences education

## Abstract

Generative artificial intelligence (AI), especially large language models, is playing an expanding role in shaping learning within health sciences education. Current discussions often focus on efficiency or academic integrity, with less attention to how learners engage with AI across evolving cognitive and developmental stages. This Perspective conceptualizes generative AI as a cognitive co-learner, an interactive system that supports idea generation, organization, and reasoning while requiring human oversight for validation and interpretation. We propose a developmental framework for AI literacy that describes stage-typical patterns of AI engagement across educational contexts characterized by differing cognitive and epistemic demands. The proposed progression from exploration to synthesis to calibration reflects changes in the functional role of AI, learner cognitive engagement, and trust in AI-generated outputs. This progression is intended as a theoretical proposition rather than an empirically validated development trajectory. Central to this framework is calibrated trust, defined as the alignment between confidence in AI outputs and their actual reliability. Across all stages, human judgment, critical evaluation, and metacognitive awareness remain essential. This Perspective highlights the need for stage-appropriate educational strategies and learning activities that support responsible AI use while strengthening health sciences education and maintaining the primacy of human cognition.

## 1 Introduction

Generative artificial intelligence (AI), particularly large language models such as ChatGPT, has become a prominent influence in education, shaping how students engage with learning across diverse contexts, including health sciences (Dwivedi et al., 2023; Kasneci et al., 2023). These systems are increasingly used to explain complex concepts, summarize information, and support problem-solving, becoming embedded in everyday study practices. Despite its widespread adoption, discussions of generative AI have largely focused on its educational opportunities, efficiency, and risks to academic integrity, with comparatively less attention to how learners engage with AI across evolving cognitive and developmental stages. Existing AI literacy frameworks, including those proposed by Long and Magerko (2020) and Ng et al. (2021), emphasize foundational competencies, knowledge, skills, and ethical awareness. While these frameworks provide important conceptual foundations, they place less emphasis on how AI engagement may evolve across educational contexts characterized by increasing cognitive and epistemic demands, particularly in health sciences education. This Perspective situates generative AI within human-AI interaction and cognitive systems, emphasizing its role in shaping how knowledge is generated, structured, and evaluated.

In response, this Perspective presents generative AI as a cognitive co-learner that supports, but does not replace, human thinking. It introduces a conceptual developmental framework for AI literacy in health sciences education in which the proposed progression is primarily organized according to increasing cognitive and epistemic demands. Pre-university, undergraduate public health, and undergraduate medical education are presented as illustrative educational contexts that exemplify differing cognitive and epistemic demands rather than as determinants of the proposed progression. The framework describes stage-typical patterns of AI engagement organized as a progression from exploration to synthesis to calibration while maintaining the central role of human judgment, critical evaluation, and ethical awareness. By integrating conceptual analysis with illustrative learner perspectives, this Perspective aims to advance understanding of generative AI as a cognitive co-learner and proposes a conceptual framework of AI literacy that progresses from exploration to synthesis to calibration. These contributions aim to inform future research, curriculum design, and the appropriate use of AI in health sciences learning.

## 2 Generative AI as a cognitive co-learner

### 2.1 Moving beyond AI as a passive tool

Generative AI, such as large language models including ChatGPT, is becoming increasingly integrated into educational settings, where it is often discussed in terms of both opportunities and risks, including efficiency and issues related to academic integrity (Dwivedi et al., 2023; Holmes et al., 2019; Kasneci et al., 2023). However, existing discussions often conceptualize generative AI primarily as an instructional instrument or assistive technology, potentially overlooking its fundamentally interactive and generative role in learning. Rather than simply supporting learning tasks, generative AI actively produces explanations, reorganizes knowledge, and adapts responses to user prompts, thereby shaping how learners engage with content and participate in the co-construction of knowledge (Tran et al., 2025). Emerging evidence indicates that students are already incorporating AI into their learning in ways that influence comprehension, problem-solving, and study behaviors (Kasneci et al., 2023; Vieriu and Petrea, 2025). This shift suggests that generative AI may function as an interactive cognitive partner that supports learning through iterative human-AI interaction while preserving human epistemic authority (Tran et al., 2025).

### 2.2 Defining AI as a cognitive co-learner

Within this evolving landscape, we conceptualize generative AI as a cognitive co-learner, an interactive system that contributes to learning by generating, structuring, and refining ideas in response to human input, while requiring human oversight for validation and contextual interpretation. Unlike conceptualizations that primarily frame as AI as an instructional instrument or assistive technology, the cognitive co-learner perspective emphasizes iterative, bidirectional interaction that shapes learner cognition over time. This perspective moves beyond viewing AI as a static tool by highlighting its role in supporting cognitive functions such as generating ideas, organizing information, and elaborating concepts, and aligns with emerging views that position AI as a collaborator in human-AI learning and cognitive support rather than simply a passive system (Lu et al., 2025; Zaim et al., 2025). While conceptually related to descriptions of AI as a collaborator in human-AI learning (Lu et al., 2025), a “more knowledgeable other” (Tran et al., 2025), or a cognitive co-pilot (Zaim et al., 2025), the cognitive co-learner perspective is situated within a developmental framework that emphasizes how learner engagement with AI, cognitive engagement, and trust evolve across stages of learning. This developmental focus underpins the proposed progression from exploration to synthesis to calibration. Accordingly, the proposed cognitive co-learner perspective complements these existing conceptualizations by emphasizing how AI engagement, cognitive engagement, and calibrated trust may vary across educational contexts characterized by increasing cognitive and epistemic demands.

Importantly, the term cognitive co-learner is used conceptually to describe AI’s interactive role in supporting human learning rather than implying that AI itself learns during the interaction. Unlike human learners, generative AI does not autonomously learn or update its underlying model during interactions with individual learners. Instead, it functions as a responsive cognitive partner that facilitates exploration, synthesis, reflection, and critical evaluation while the learner retains epistemic authority and responsibility for knowledge construction. Consistent with this limitation, generative AI systems generate responses based on learned statistical patterns in language and may produce convincing but inaccurate information without human validation (Cascella et al., 2023; Gilson et al., 2023; Kung et al., 2023). As such, conceptualizing generative AI as a cognitive co-learner highlights its role as an interactive partner in learning, while reaffirming the centrality of human judgment in guiding and validating its contributions. In this Perspective, calibrated trust refers to the alignment between a learner’s confidence in AI-generated outputs and their actual reliability. This conceptualization builds on the broader literature on appropriate reliance in trust in automation, which emphasizes that trust should be proportional to system capabilities and reliability (Lee and See, 2004). Miscalibration may occur as over-trust (misuse), where AI-generated outputs are accepted without sufficient verification, or under-trust (disuse), where potentially useful AI support is unnecessarily rejected (Parasuraman and Riley, 1997). Accordingly, calibrated trust involves recognizing when AI can appropriately support learning and when independent verification and human judgment are required.

### 2.3 Human-AI collaborative cognition

Viewing AI as a cognitive co-learner reframes learning as a collaborative and iterative process between human and machine. Learners engage in cycles of prompting, receiving outputs, questioning, and refining their understanding, resulting in a more interactive and dialogic form of knowledge construction (Lu et al., 2025; Zaim et al., 2025). This interaction may support higher-order cognitive engagement by encouraging learners to compare, organize, refine, and critically evaluate AI-generated information rather than accepting outputs uncritically, while also introducing risks related to over-reliance, reduced critical engagement, and superficial understanding (Cascella et al., 2023; Kasneci et al., 2023). The process reflects elements of metacognition, as learners must actively monitor, evaluate, and regulate their engagement with AI-generated information (Flavell, 1979; Kasneci et al., 2023). At the same time, reliance on AI may involve forms of cognitive offloading, where certain cognitive tasks are delegated to external systems, potentially altering how knowledge is processed and retained (Risko and Gilbert, 2016). Together, these processes provide a conceptual basis for the proposed progression from exploration to synthesis and ultimately to calibration by encouraging increasingly reflective and context-sensitive engagement with AI. These dynamics highlight that learning with AI is not a one-directional transfer of information, but a co-constructed cognitive process.

### 2.4 The central role of human oversight and judgment

Building on this conceptualization, the proposed framework emphasizes that effective AI-supported learning depends on sustained human judgment, critical evaluation, and contextual interpretation. Because AI-generated outputs may contain inaccuracies, omissions, or biases, while presenting information with unwarranted confidence (Alkaissi and McFarlane, 2023; Cascella et al., 2023; Gilson et al., 2023), learners remain responsible for verifying information, interpreting meaning, and making informed decisions about the appropriate use of AI-generated content (Kasneci et al., 2023; Kung et al., 2023). This highlights the importance of developing AI literacy as a metacognitive competence, involving not only technical proficiency but also the capacity to critically evaluate and appropriately rely on AI-generated outputs. In this context, AI functions as a cognitive co-learner that augments but does not replace human cognition, with final epistemic authority residing with the learner.

## 3 A developmental and discipline-sensitive framework

We conceptualize AI literacy as a developmental process primarily shaped by increasing cognitive and epistemic demands. The illustrative educational contexts presented in this Perspective (pre-university, undergraduate public health, and undergraduate medical education) serve as examples of how these differing demands may be expressed in practice rather than as determinants of the proposed progression. While the illustrative learner perspectives focus primarily on public health and medical education, the framework is intended to highlight how AI engagement may vary across educational settings and disciplinary contexts characterized by different cognitive and epistemic demands. This perspective aligns with constructivist and metacognitive theories of learning, which emphasize active knowledge construction and self-regulation (Flavell, 1979; Vygotsky, 1978), while extending them by conceptualizing generative AI as an interactive cognitive partner that influences how learners organize information, evaluate knowledge, and calibrate trust in AI-generated outputs.

Rather than a fixed set of technical skills, AI literacy reflects how learners interact with, interpret, and evaluate AI-generated outputs in relation to their own thinking processes. As learners progress, engagement with generative AI shifts from exploratory use and emerging trust to structured synthesis of complex information, and ultimately to calibrated reasoning in context-sensitive situations. This proposed progression represents a conceptual model of stage-typical patterns of AI engagement across educational contexts characterized by increasing cognitive and epistemic demands, rather than an empirically validated developmental trajectory or individual learners. To illustrate these stage-specific interactions, we present brief learner perspectives as conceptual exemplars based on lived learning experiences across stages of training. These narratives are intended solely as illustrative examples to demonstrate how learners may engage with generative AI across different educational contexts and stages of development. They do not represent empirical data, qualitative findings, or systematically collected observations, but rather serve as conceptual illustrations that broadly align with emerging literature on AI-supported learning behaviors.

**Figure 1:**
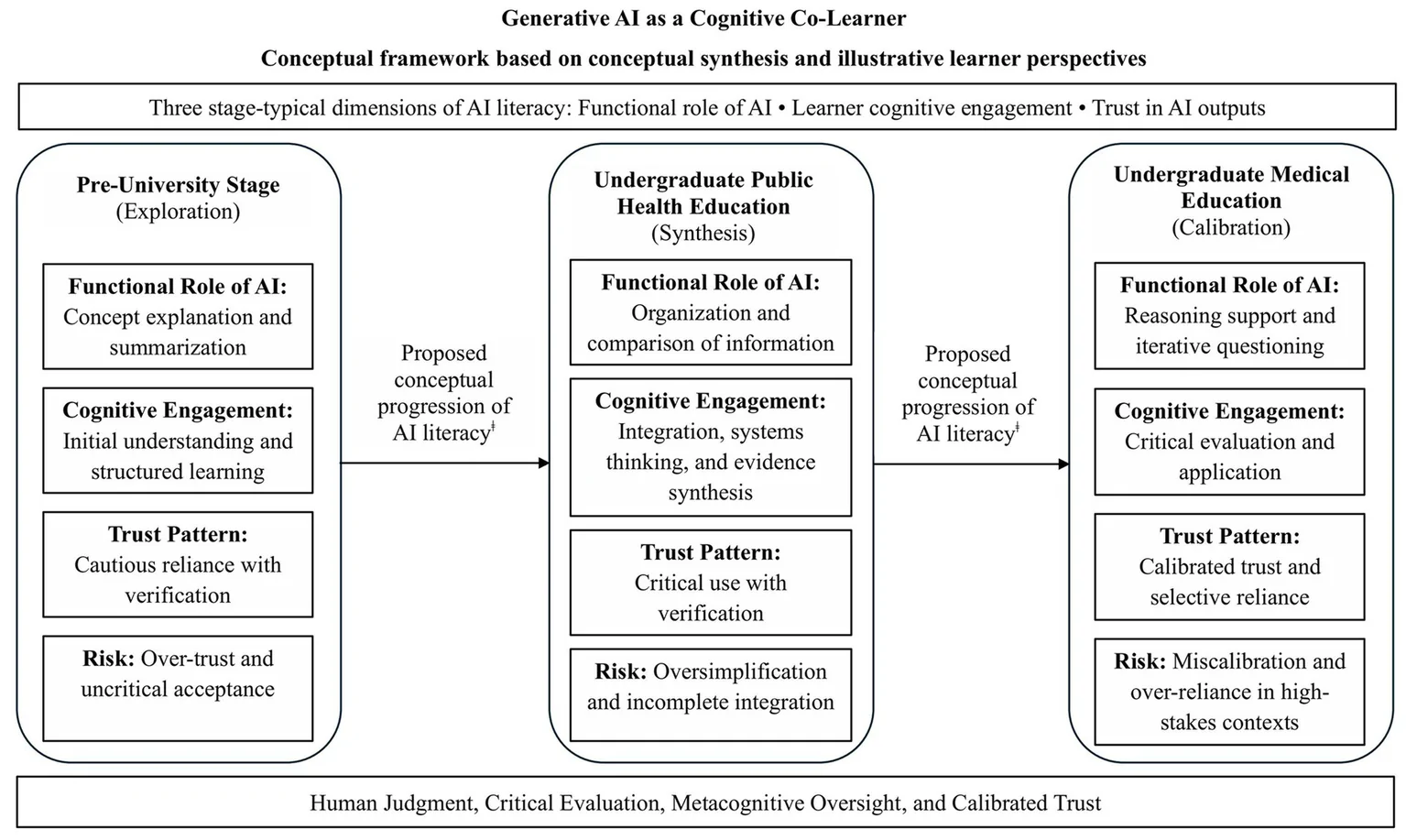
A conceptual developmental framework of generative AI as a cognitive co-learner in health sciences education. The framework illustrates stage-typical patterns of AI engagement from exploration to synthesis to calibration across illustrative educational contexts characterized by increasing cognitive and epistemic demands. Across all stages, human judgment, critical evaluation metacognitive oversight, and calibrated trust underpin responsible AI use. ^‡^The proposed progression reflects increasing cognitive and epistemic demands and represents a conceptual model rather than a fixed, universal, or empirically validated developmental sequence.

The proposed framework was developed through a conceptual synthesis of the literature on AI-supported learning and human-AI interaction, together with reflection on the illustrative learner perspectives presented in this article. Drawing on this conceptual synthesis and reflected in the illustrative learner perspectives, three recurring dimensions were identified: the functional role of AI, learner cognitive engagement, and trust in AI-generated outputs. In addition, stage-specific risks associated with AI use were identified at each stage. Comparison across the conceptual literature and the illustrative educational contexts suggested a progression from exploration to synthesis to calibration, reflecting increasing cognitive and epistemic demands. These recurring patterns informed the development of the framework presented in Figure 1.

## 4 Illustrative learner perspectives as conceptual exemplars across developmental stages

### 4.1 Pre-university stage: trust formation and early learning habits

The following perspective is presented as a conceptual illustration rather than an empirical account. At the pre-university stage, learners often encounter generative AI for the first time, and early patterns of use begin to shape both epistemic trust and study habits. Initial engagement is typically exploratory, with AI used to simplify complex concepts and support comprehension when traditional materials feel overwhelming. In practice, learners may use generative AI systems, such as ChatGPT, to generate summaries or explanations, making difficult content more accessible while also revealing limitations, including occasional inaccuracies or incomplete information. This encourages the development of cautious practices, such as verifying important content using reliable sources.

Over time, AI becomes integrated into study routines as a tool for structuring understanding. For example, generating bullet-point summaries before reading full texts can create a preliminary framework that supports deeper engagement, while AI-assisted outlining and AI-supported literature organization tools, such as NotebookLM, may help manage multiple sources during writing. Importantly, AI is not treated as a source of final answers, but as a learning support that facilitates understanding while requiring independent judgment. These early experiences illustrate how initial exposure to AI shapes both trust calibration and learning behaviors, with implications for later stages of education.

### 4.2 Undergraduate public health: systems thinking and evidence synthesis

At the undergraduate level in public health, learning involves engaging with complex systems and integrating evidence from multiple sources. In this context, systems thinking involves understanding how multiple determinants, health system components, and public health processes interact to influence population health outcomes. For example, learners may use generative AI systems and AI-assisted learning tools, including platforms such as Google Gemini, ChatGPT, NotebookLM, and QuillBot, to support different stages of this process. They are often used to generate initial overviews of new topics, helping to quickly understand structure before engaging more deeply with textbooks, lecture materials, and official sources. For instance, when preparing presentations on communicable diseases, AI-assisted summaries help organize key concepts such as transmission, prevention, and health system responses, making it easier to understand how these elements interact within broader systems.

As learning progresses, AI-assisted literature organization tools, such as NotebookLM, may support comparison across multiple articles and identification of recurring themes, facilitating evidence synthesis and management of large amounts of information. However, limitations are also recognized: AI outputs may be oversimplified, incomplete, or presented with unwarranted confidence. As a result, verification using peer-reviewed literature and authoritative guidelines remains essential. AI is therefore used as a starting point for organizing information and guiding further inquiry, while critical appraisal, interpretation, and judgment remain central to effective learning.

### 4.3 Undergraduate medical education: calibration and clinical reasoning

In undergraduate medical education, learning occurs in high-stakes contexts that require accurate reasoning and application of knowledge. At this stage, for example, learners may use large language models, including Claude and ChatGPT, to help manage the volume and complexity of biomedical information. They are used to restructure content into more accessible formats and to generate explanations at different levels of depth, supporting both foundational understanding and clinical application. AI-generated notes and clinically oriented question banks are also used to test comprehension and promote active learning beyond memorization.

AI outputs, however, are not accepted uncritically. They are compared with lecture materials, textbooks, and research articles to ensure accuracy, with inconsistencies prompting iterative questioning, correction, and challenge of AI outputs. This iterative interaction supports the development of metacognitive awareness, particularly in recognizing when AI outputs can be trusted and when they require closer scrutiny. AI functions as a learning support that facilitates reasoning, while responsibility for interpretation and verification remains with the learner. This reflects a more advanced stage of AI literacy, characterized by calibrated trust and the need to balance AI support with independent clinical judgment.

### 4.4 Developmental progression across stages

Across the illustrative learner perspectives, consistent patterns emerged in the functional role of AI, learner cognitive engagement, trust in AI-generated outputs, and stage-specific risks. These recurring patterns formed the conceptual basis of the proposed framework. In this context, “developmental” refers to increasing cognitive and epistemic demands rather than strictly age-based progression. These patterns are conceptual and may vary across contexts, disciplines, and levels of training.

Three dimensions define this progression. First, the functional role of AI shifts from concept explanation to information organization and synthesis, and then to reasoning support and iterative questioning. Second, cognitive engagement moves from initial understanding to integration of multiple sources, and then to critical evaluation and application. Third, trust in AI outputs develops from cautious verification to more deliberate calibration, with selective reliance alongside independent judgment. Together, these dimensions form the basis of a conceptual progression from exploration to synthesis to calibration. Although these dimensions are presented together for conceptual clarity, they may not always develop in parallel across learners or educational contexts, reflecting differences in cognitive and epistemic demands and individual learning experiences. This progression represents stage-typical patterns of AI engagement across the illustrative educational contexts presented in the Perspective and should not be interpreted as empirical evidence that individual learners necessarily progress through these stages over time.

This proposed progression is primarily organized according to increasing cognitive and epistemic demands, while the educational contexts presented in this Perspective serve as illustrative examples of how these demands may be expressed in different learning environments rather than as the primary drivers of the framework. At the pre-university stage, engagement is primarily exploratory, with emerging trust and study habits. In undergraduate public health, AI supports systems thinking and evidence synthesis. In medical education, AI use becomes more context-sensitive and high-stakes, requiring calibrated trust and application in reasoning and decision-making. Although the illustrative educational contexts reflect increasing cognitive and epistemic demands, the proposed framework is intended to represent a conceptual pattern of AI engagement rather than a discipline-specific or empirically validated developmental sequence.

Across all stages, AI supports learning but does not replace human judgment, critical evaluation, or contextual interpretation. This framing highlights the need for stage-appropriate strategies that support effective AI use and responsible engagement. The proposed framework is illustrated in Figure 1.

## 5 Ethical, cognitive, and educational risks

The integration of generative AI in health sciences education introduces important cognitive, epistemic, and ethical risks. These risks may manifest differently across the proposed stages of AI literacy and highlight the importance of calibrated trust, metacognitive oversight, and human judgment throughout the developmental framework. Excessive dependence on AI may lead to cognitive offloading, potentially diminishing deep learning and independent reasoning (Gerlich, 2025; Risko and Gilbert, 2016). Excessive cognitive offloading may also undermine the intended role of generative AI as a cognitive co-learner by reducing opportunities for active reasoning, reflection, and independent knowledge construction. In addition, AI outputs are not inherently reliable and may include bias, hallucinations, or incomplete information, often presented with unwarranted confidence (Alkaissi and McFarlane, 2023; Gilson et al., 2023). These limitations contribute to epistemic shifts, where learners may increasingly rely on AI as a source of knowledge, raising questions about trust and authority in learning (Kasneci et al., 2023; Lu et al., 2025). Unequal access to AI tools and differences in AI literacy may exacerbate educational inequities, particularly as emerging technologies risk reinforcing existing digital divides (Beckman et al., 2025; Kasneci et al., 2023). These disparities may also influence learners’ opportunities to develop AI literacy, potentially shaping how they engage with the stage-typical patterns proposed in this framework. Furthermore, the integration of AI introduces concerns about academic integrity and responsible use, particularly as the boundaries between acceptable assistance and misuse become less clear (Dwivedi et al., 2023). These challenges highlight the need for critical evaluation, ethical awareness, and human oversight so that AI contributes to, rather than detracts from, effective learning (Floridi et al., 2018; Thaichana et al., 2025).

## 6 Discussion

This Perspective proposes a conceptual framework that views generative AI as a cognitive co-learner in health sciences education, framing AI literacy through stage-typical patterns of AI engagement across educational contexts characterized by increasing cognitive and epistemic demands. Existing literature has examined the potential benefits and risks associated with generative AI, including efficiency, issues related to academic integrity, misuse, and the need for critical evaluation (Dwivedi et al., 2023; Kasneci et al., 2023). Building on these perspectives, the present framework conceptualizes AI-supported learning through stage-typical patterns of engagement progressing conceptually from exploration to synthesis to calibration. This progression represents a theoretical proposition rather than an empirically validated developmental trajectory.

At earlier stages, AI supports concept clarification and structured learning, consistent with evidence that students use generative AI to simplify complex material and support comprehension (Kasneci et al., 2023; Vieriu and Petrea, 2025). Across education contexts characterized by increasing cognitive and epistemic demands, AI engagement may shift from exploratory use to structured synthesis and ultimately to calibrated reasoning in context-sensitive situations. This conceptualization is consistent with recent literature describing generative AI as supporting collaborative human-AI learning and higher-order cognitive engagement (Lu et al., 2025; Zaim et al., 2025). At more advanced stages, particularly in medical education, AI may support reasoning while requiring critical evaluation in high-stakes contexts (Cascella et al., 2023; Kung et al., 2023). This progression reflects increasing cognitive and epistemic demands, highlighting the importance of metacognitive monitoring, critical evaluation, and regulation in AI-supported learning (Flavell, 1979).

The framework emphasizes calibrated trust as a central component of effective human-AI interaction, building on the broader literature on appropriate trust in automation while extending these concepts to AI-supported learning in health sciences education (Lee and See, 2004; Parasuraman and Riley, 1997). Across the proposed stages, appropriate reliance on AI involves aligning learner confidence with the reliability of AI-generated outputs, while avoiding both over-trust and under-trust. This conceptualization is consistent with documented concerns regarding AI-generated hallucinations and potentially misleading outputs (Alkaissi and McFarlane, 2023; Gilson et al., 2023), highlighting that appropriate trust requires ongoing critical evaluation rather than unquestioning reliance. This conceptualization is also consistent with recent literature describing generative AI as supporting collaborative human-AI learning and higher-order cognitive engagement (Lu et al., 2025; Zaim et al., 2025). Importantly, calibrated trust reinforces that AI outputs should not be treated as epistemically authoritative sources, with responsibility for validation remaining with the learner. This shift is embedded in iterative human-AI interaction, reflecting a dialogic co-learning process (Lu et al., 2025).

These interactions may involve cognitive offloading, as learners delegate tasks to AI systems (Risko and Gilbert, 2016). While such offloading may enhance efficiency, it may reduce depth of understanding and promote superficial engagement if not critically managed. Together, these dynamics position generative AI as a cognitive aid while also presenting a potential risk of reduced cognitive engagement, depending on how it is integrated into learning practices.

This conceptual perspective reflects the differing cognitive and epistemic demands encountered across health sciences education. Within this context, generative AI may support learning in different ways, ranging from conceptual understanding to context-sensitive reasoning, while requiring rigorous verification and interpretation (Cascella et al., 2023; Kung et al., 2023). This highlights that AI engagement may vary across disciplinary and educational contexts rather than being uniform across learning environments. Although the illustrative examples focus on public health and medical education, the framework may be applicable to other health sciences disciplines that involve differing cognitive and epistemic demands. Future work should examine its relevance across a broader range of educational contexts. The proposed framework also generates testable propositions, including whether learner engagement, cognitive engagement, and calibrated trust develop as stage-typical patterns across educational contexts characterized by differing cognitive and epistemic demands. Future research is also needed to develop practical approaches for identifying learner stages, assessing calibrated trust, and informing curriculum design and educational practice.

Importantly, the proposed framework should be interpreted as a conceptual model rather than evidence of developmental change and should not be interpreted as a prescriptive or universal sequence of AI literacy development. The illustrative learner perspectives represent different individuals across educational contexts rather than longitudinal observations of the same learners. Consequently, the proposed progression from exploration to synthesis to calibration should be regarded as a theoretical proposition that warrants future empirical evaluation, particularly through longitudinal and mixed-methods research.

Across all stages, the framework emphasizes human judgment, critical evaluation, and metacognitive oversight, consistent with ethical perspectives that maintain human responsibility in decision-making (Floridi et al., 2018). Although generative AI can improve access to information and facilitate learning, learners still need to interpret and apply knowledge appropriately, especially in high-stakes contexts. This Perspective conceptualizes AI literacy as a context-dependent capability that complements existing AI literacy frameworks by emphasizing effective human-AI interaction, human judgment, critical evaluation, and calibrated trust across educational contexts.

## 7 Implications for health sciences education

This Perspective suggests that AI literacy in health sciences education should be approached as a developmental and context-sensitive competency shaped through human-AI interaction. Educational strategies may be aligned with stages of training, with early learning emphasizing guided use, verification, and foundational understanding, for example, by encouraging learners to compare AI-generated explanations with trusted educational resources. At intermediate stages, AI may support evidence synthesis and critical comparison of multiple information sources, whereas more advanced stages may emphasize context-sensitive reasoning and critical appraisal of AI-generated outputs in complex or high-stakes situations. Across stages, supporting metacognitive monitoring and calibrated trust is important, enabling learners to recognize the strengths and limitations of AI-generated outputs. Given the epistemic demands of health sciences, curricula should reinforce the primacy of human judgment, particularly in high-stakes contexts where interpretation and decision-making cannot be delegated to AI. Although the proposed framework is conceptual and not intended as a formal assessment model, it may provide a foundation for curriculum design and educational planning by aligning AI-supported learning activities with increasing cognitive and epistemic demands. It may also inform the future development of AI literacy curricula, educational resources, and assessment approaches that promote calibrated trust and responsible human-AI interaction. Overall, this framework highlights the need to integrate generative AI in ways that support learning while maintaining critical engagement, responsible use, and appropriate reliance.

## 8 Conclusion

Generative AI can act as a cognitive co-learner that supports, but does not replace, human thinking. This Perspective proposes a conceptual framework in which AI literacy is represented through stage-typical patterns of AI engagement progressing conceptually from exploration to synthesis to calibration across educational contexts characterized by increasing cognitive and epistemic demands. Within this conceptual progression, calibrated trust, human judgment, critical evaluation, and metacognitive oversight remain essential for interpreting, verifying, and appropriately applying AI-generated outputs. This perspective emphasizes the need to integrate AI in a manner that supports learning while maintaining critical evaluation and responsible use in health sciences education.

## Data Availability

The original contributions presented in the study are included in the article/supplementary material, further inquiries can be directed to the corresponding author.

## References

[ref1] Alkaissi H. McFarlane S. I. (2023). Artificial hallucinations in ChatGPT: implications in scientific writing. Cureus 15:e35179. doi: 10.7759/cureus.35179, 36811129 PMC9939079

[ref2] Beckman K. Apps T. Howard S. K. Rogerson C. Rogerson A. Tondeur J. (2025). The GenAI divide among university students: a call for action. Internet High. Educ. 67:101036. doi: 10.1016/j.iheduc.2025.101036

[ref3] Cascella M. Montomoli J. Bellini V. Bignami E. (2023). Evaluating the feasibility of ChatGPT in healthcare: an analysis of multiple clinical and research scenarios. J. Med. Syst. 47:33. doi: 10.1007/s10916-023-01925-4, 36869927 PMC9985086

[ref4] Dwivedi Y. K. Kshetri N. Hughes L. Slade E. L. Jeyaraj A. Kar A. K. . (2023). Opinion paper: “so what if ChatGPT wrote it?” multidisciplinary perspectives on opportunities, challenges and implications of generative conversational AI for research, practice and policy. Int. J. Inf. Manag. 71:102642. doi: 10.1016/j.ijinfomgt.2023.102642

[ref5] Flavell J. H. (1979). Metacognition and cognitive monitoring: a new area of cognitive–developmental inquiry. Am. Psychol. 34, 906–911. doi: 10.1037/0003-066X.34.10.906

[ref6] Floridi L. Cowls J. Beltrametti M. Chatila R. Chazerand P. Dignum V. . (2018). AI4People-an ethical framework for a good AI society: opportunities, risks, principles, and recommendations. Minds Mach (Dordr) 28, 689–707. doi: 10.1007/s11023-018-9482-5, 30930541 PMC6404626

[ref7] Gerlich M. (2025). AI tools in society: impacts on cognitive offloading and the future of critical thinking. Societies 15:6. doi: 10.3390/soc15010006

[ref8] Gilson A. Safranek C. W. Huang T. Socrates V. Chi L. Taylor R. . (2023). How does ChatGPT perform on the United States medical licensing examination (USMLE)? The implications of large language models for medical education and knowledge assessment. JMIR Med. Educ. 9:e45312. doi: 10.2196/45312 36753318 PMC9947764

[ref9] Holmes W. Bialik M. Fadel C. (2019). Artificial Intelligence in Education. Promise and Implications for Teaching and Learning. Center for Curriculum Redesign. Available online at: https://circls.org/primers/artificial-intelligence-in-education-promises-and-implications-for-teaching-and-learning

[ref10] Kasneci E. Sessler K. Küchemann S. Bannert M. Dementieva D. Fischer F. . (2023). ChatGPT for good? On opportunities and challenges of large language models for education. Learn. Individ. Differ. 103:102274. doi: 10.1016/j.lindif.2023.102274

[ref11] Kung T. H. Cheatham M. Medenilla A. Sillos C. De Leon L. Elepaño C. . (2023). Performance of ChatGPT on USMLE: potential for AI-assisted medical education using large language models. PLOS Digit Health 2:e0000198. doi: 10.1371/journal.pdig.0000198, 36812645 PMC9931230

[ref12] Lee J. D. See K. A. (2004). Trust in automation: designing for appropriate reliance. Hum. Fact. 46, 50–80. doi: 10.1518/hfes.46.1.50_30392, 15151155

[ref13] Long D. Magerko B. (2020). What is AI literacy? Competencies and design considerations. Proceedings of the 2020 CHI Conference on Human Factors in Computing Systems,

[ref14] Lu J. Yan Y. Huang K. Yin M. Zhang F. (2025). Do we learn from each other: understanding the human-AI co-learning process embedded in human-AI collaboration. Group Decis. Negot. 34, 235–271. doi: 10.1007/s10726-024-09912-x

[ref15] Ng D. T. K. Leung J. K. L. Chu S. K. W. Qiao M. S. (2021). Conceptualizing AI literacy: an exploratory review. Comput. Educ. 2:100041. doi: 10.1016/j.caeai.2021.100041, 38826717

[ref16] Parasuraman R. Riley V. (1997). Humans and automation: use, misuse, disuse, abuse. Hum. Factors 39, 230–253. doi: 10.1518/00187209777854388

[ref17] Risko E. F. Gilbert S. J. (2016). Cognitive offloading. Trends Cogn. Sci. 20, 676–688. doi: 10.1016/j.tics.2016.07.002, 27542527

[ref18] Thaichana P. Oo M. Z. Thorup G. L. Chansakaow C. Arworn S. Rerkasem K. (2025). Integrating artificial intelligence in medical writing: balancing technological innovation and human expertise, with practical applications in lower extremity wounds care. Int J Low Extrem Wounds 25, 407–413. doi: 10.1177/15347346241312814, 39801150

[ref19] Tran M. Balasooriya C. Semmler C. Rhee J. (2025). Generative artificial intelligence: the 'more knowledgeable other' in a social constructivist framework of medical education. NPJ Digit. Med. 8:430. doi: 10.1038/s41746-025-01823-8, 40646156 PMC12254308

[ref20] Vieriu A. M. Petrea G. (2025). The impact of artificial intelligence (AI) on students’ academic development. Educ. Sci. 15:343. doi: 10.3390/educsci15030343

[ref21] Vygotsky L. S. (1978). Mind in Society: Development of Higher Psychological Processes. Cambridge, MA, USA: Harvard University Press.

[ref22] Zaim M. Arsyad S. Waluyo B. Ardi H. Al Hafizh M. Zakiyah M. . (2025). Generative AI as a cognitive co-pilot in English language learning in higher education. Educ. Sci. 15:686. doi: 10.3390/educsci15060686

